# Eosinophilic Fasciitis: A Rare Skin Sclerosis

**DOI:** 10.4061/2011/716935

**Published:** 2010-12-01

**Authors:** Amandine Servy, Thierry Clérici, Caroline Malines, Jean-Marie Le Parc, Jean-François Côté

**Affiliations:** ^1^Service d'Anatomie et Cytologie Pathologiques, Hôpital Ambroise Paré, AP-HP, Université de Versailles Saint-Quentin-en-Yvelines, 9 Avenue Charles de Gaulle, 92104 Boulogne-Billancourt Cedex, France; ^2^Service de Rhumatologie, Hôpital Ambroise Paré, AP-HP, Université de Versailles Saint-Quentin-en-Yvelines, 9 Avenue Charles de Gaulle, 92104 Boulogne-Billancourt Cedex, France

## Abstract

Eosinophilic fasciitis (Schulman's syndrome) is a rare disease with specific clinical symptoms such as the groove sign which facilitate diagnosis. We report a typical case of eosinophilic fasciitis in an otherwise healthy 49-year-old man who presented with “prayer and groove signs”. Histological analysis showed sclerosis and eosinophilic infiltration of the fascia. The patient was successfully treated with systemic corticotherapy and Cyclosporine. A short review of the clinicopathological features of the lesions is presented.

## 1. Case Report

An otherwise healthy 49-year-old man presented with a history of several months of severe fatigue with myalgia and polyarthritis, rapidly worsening with painful swelling of legs and hands, joint stiffness, and thickening of the skin. He took neither medication nor toxic oil.

**Figure 1:**
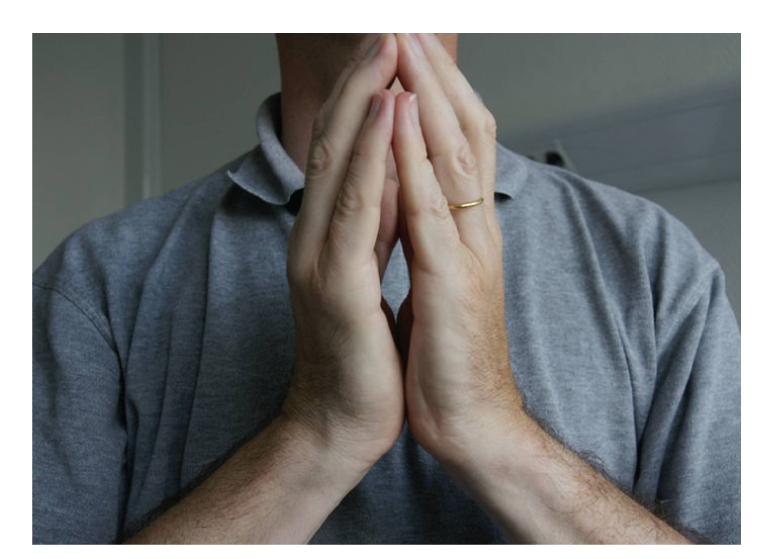
Prayer sign due to hand tenosynovitis.

**Figure 2:**
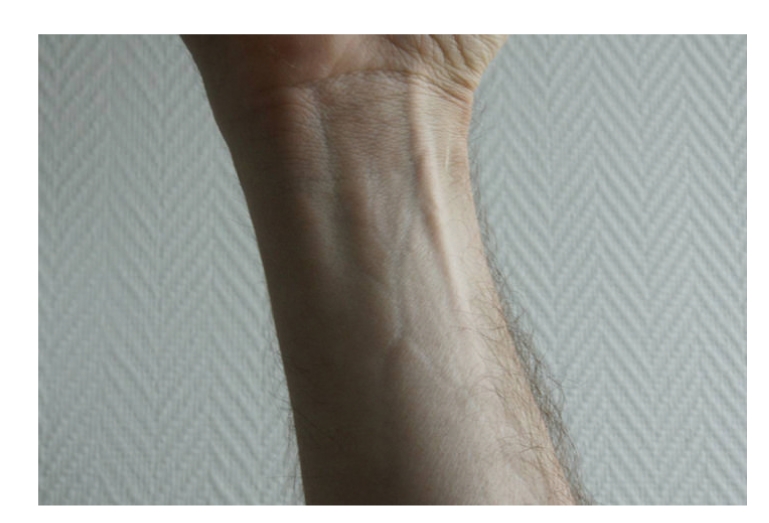
Wrist with venous furrowing or the “groove sign”.

Physical examination revealed symmetrical lesions of dermal sclerosis with normal superficial skin. Prayer sign due to tenosynovitis of the hand (Figure 1), venous furrowing (“groove sign” or “le signe du canyon” in France, Figure 2) of the wrist, and arthritis in the feet and knees were noted. We saw no telangiectasia, calcinosis, megacapillary, sclerodactyly, or mucosal involvement. The patient showed neither Raynaud phenomenon nor digital ulceration. 

The capillary microscopy exam showed pericapillary oedema and capillary rarefaction corresponding to a nonspecific microangiopathy.

Laboratory results showed hypereosinophilia (2063/mm^3^) with an elevated C-reactive protein level (23 mg/L), transitory leucocytosis, and oligoclonal hypergammaglobulinemia (IgG kappa and IgM lambda levels not measurable). Immunological results were normal or negative for antinuclear antibodies, extractable nuclear antigens, anticitrullinated peptides, rheumatoid and complement factor. Stools were normal, and the patient had received antiparasite treatment (Ivermectine 1 day). The computed tomography scan of the chest, abdomen, and pelvis was normal. Magnetic resonance imaging (MRI) of the pelvis was normal at the time of the initial symptoms and was not repeated. 

**Figure 3:**
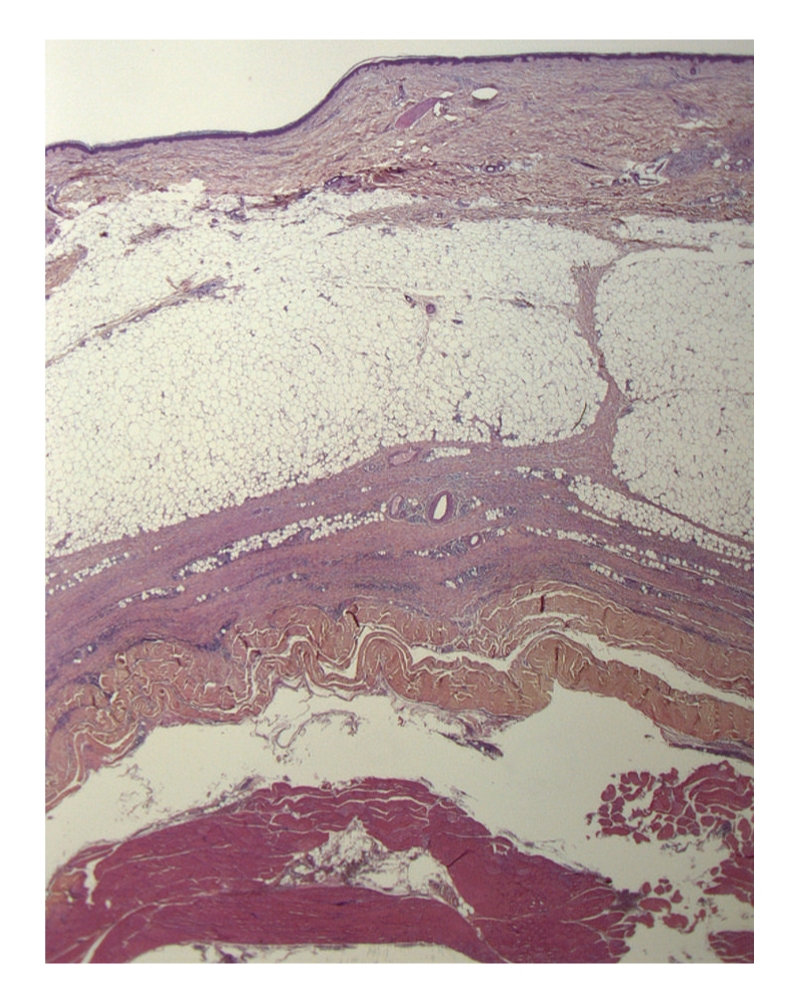
Histologic findings show thickening of the interlobular septa of panniculus.

**Figure 4:**
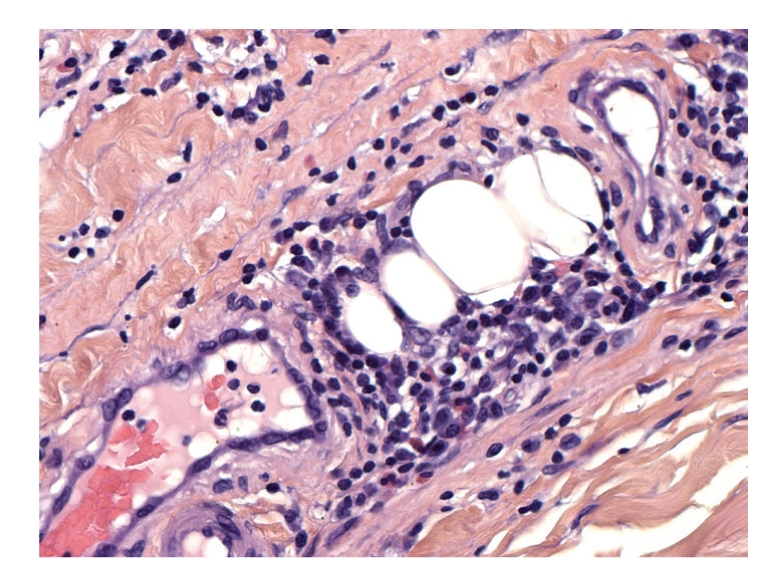
Fascial sclerosis with lymphocytic, plasma cell, and eosinophilic infiltration.

A deep surgical biopsy of the left leg was performed. The histological findings are shown in Figures 3 and 4. 

The diagnosis of eosinophilic fasciitis (Schulman's syndrome) was done.

## 2. Microscopic Findings and Clinical Course

The surgical biopsy included skin, fascia, and muscle, confirmed the diagnosis of eosinophilic fasciitis. Histological analysis showed fascial sclerosis, lymphocytic, plasma cell, and eosinophilic infiltration of the fascia, and muscle and interlobular septae of panniculus. Thickening of the interlobular septa of panniculus was seen without dermal sclerosis (Figures 3 and 4).

The patient received systemic corticotherapy, initially 1 g of Solumedrol intravenous for two days then reduced to 100 mg per day for one week. Treatment was then changed to oral Prednisone at 60 mg with accompanying immunosuppressive therapy (Cyclosporine 150 mg/day). Within one week, joint swelling, stiffness, oedema, and laboratory abnormalities decreased. In conjunction with the same level of Cyclosporine, the Prednisone was gradually reduced to 30 mg/day for one month, then to 10 mg/day for 3 months. At 4 months, the patient completely recovered, showing no symptoms. The oral Prednisone was reduced to 3 mg/day for the following 10 months and discontinued thereafter. In the same time, Cyclosporine was reduced to 50 mg/day for 2 months, 25 mg/day for 6 months, then discontinued. At 12 months, all laboratory tests were normal.

## 3. Discussion

Eosinophilic fasciitis was described in 1975 by Shulman [1] and is regarded as a variant of scleroderma [2]. Progression to scleroderma has been documented in several circumstances [3]. Many conditions may appear similar such as hematologic disease [4], Borrelia burgdorferi infection, toxicity (oil, L-tryptophan) [5], and drug adverse event [6]. The disease appears in 40- to 50-year-old men and women with no sex bias, but it is seen earlier in men. Symmetrical and distal, mainly forearm and leg, lesions classically appear after trauma or physical effort [7]. 

The pathophysiology is unknown and unclear. Shakoory et al. [8] suggest a role for mast cells and cytokines. Abnormal circulating T-cell clones and increased interleukin-5 production could be responsible for the eosinophilia and eosinophil-mediated tissue injury as suggested by French et al. [9]. 

Dermatologic examination shows oedema, skin induration with a “peau d'orange” appearance, venous furrowing (a very specific sign), and occasionally localised morphea. Raynaud's phenomenon is unusual. Capillary microscopy is normal in 84% of cases, and systemic sclerosis is never seen [10]. Joint and muscle pain, as well as asthenia, are frequent. Joints contractions are linked to skin sclerosis, tenosynovitis (carpal tunnel syndrome and prayer sign) with muscle, and aponeurotic retraction.

Prayer sign is common (56%) [11] but nonspecific. These conditions can be seen in sclerodermiform diseases as well as cheiroarthropathy, a limited joint mobility associated with diabetes [12, 13].

The main clinical differential diagnoses are sclerodermiform diseases including systemic sclerosis with Raynaud's phenomenon, calcinosis, megacapillary, a specific pattern in capillary microscopy, and antinuclear antibodies, as well as toxic oil syndrome and eosinophilia-myalgia syndrome due to L-tryptophan intake [5]. These last 2 are clinically similar to eosinophilic fasciitis; only anamnesis can distinguish them.

Laboratory findings frequently show peripheral blood eosinophilia, elevated sedimentation rates, and hypergammaglobulinemia (IgM or IgG). Hypereosinophilia is frequently seen. The principal causes are dermatosis (atopic dermatitis, urticaria, bullous pemphigoid, dermatitis herpetiformis, and drug reaction), allergies, parasitic infection, hematologic disease, cancer, or autoimmune diseases.

MRI is a very important diagnostic tool showing highly characteristic signs: fascial thickening, hyperintense signal within the fascia on fluid-sensitive sequences, and fascial enhancement after intravenous contrast administration. MRI is useful for guided muscle biopsy [14, 15] which should be performed to confirm the diagnosis (deep skin biopsy, ideally down to muscle).

Histology shows normal epidermis, rarely atrophic. The dermis is often sclerotic with inflammatory infiltrate composed of lymphocytes, eosinophils, plasma cells, and histiocytes. Beneath the dermis, fibrotic septae are seen with the same inflammatory infiltrate, thick fascia with sclerosis, and occasionally inflammatory polymorphic infiltrate with varying numbers of eosinophils. The eosinophilic infiltrate can spread into the muscle fibers. Histopathologic differential diagnosis should rule out erythema nodosum, bite reaction, parasite reaction, and Well's syndrome. These findings are similar and sometimes indistinguishable, particularly in superficial biopsies and in cases where no clinical information is provided to the pathologist. If present, eosinophilic infiltrate in fascia and muscle is specific, contrary to superficial disorders. Thus a deep biopsy to include muscle is ideal, preferably MRI guided. 

Due to the rarity of this disease, it is difficult to evaluate the efficacy of eosinophilic fasciitis therapy. Frontline treatment is corticosteroids [16], which often induce biological normalisation and total or partial clinical response. Corticoid-sparing agents are sometimes used [17–19], as well as immunosuppressive therapy (cyclosporine, cyclophosphamide, and azathioprine), hydroxychloroquine, dapsone, or immunoglobulins effectively, alone or combined. These options, however, may be contraindicated by poor response or adverse effects.

##  Conflict of interests

No conflict of interests is pertaining to our paper.

##  Ethical Guidelines

No human or animal experiments were performed in this work.
